# Pyroptosis-driven immune dysregulation in COPD: molecular mechanisms and therapeutic implications

**DOI:** 10.3389/fimmu.2025.1686175

**Published:** 2025-12-16

**Authors:** Feng-Xian Ni, Hui-Xian Wang, Jie Hu, Pei-Sheng Chen, Hua-Jing Huang, Hui-Hui Chen, Dong-Hui Huang, Ze-Bo Jiang

**Affiliations:** 1 Zhuhai Hospital of Integrated Traditional Chinese & Western Medicine, Zhuhai, Guangdong, China; 2 Department of Respiratory Medicine, Guangdong Provincial Hospital of Traditional Chinese Medicine Zhuhai Branch, Guangdong, China; 3 Northeastern University, Boston, MA, United States

**Keywords:** chronic obstructive pulmonary disease, pyroptosis, GSDMD, immune responses, inflammasome, therapeutic targeting

## Abstract

Pyroptosis, a programmed cell death mechanism mediated by gasdermin proteins such as GSDMD, is typically activated by inflammasomes. While essential for host defense against infections, excessive pyroptosis contributes to chronic inflammation and exacerbates inflammatory diseases. In chronic obstructive pulmonary disease (COPD), dysregulated pyroptosis interacts with immune cells—including neutrophils, macrophages, and T lymphocytes—to perpetuate inflammation, tissue damage, and acute exacerbations. This review explores the molecular mechanisms of pyroptosis, its cell-type-specific roles in COPD pathogenesis, and its implications for therapeutic targeting. By synthesizing evidence from primary research, we highlight how pyroptosis influences immune dysregulation in COPD and propose novel strategies for disease management.

## 1 Introduction

Chronic obstructive pulmonary disease (COPD) represents a major and escalating global health challenge, characterized by persistent respiratory symptoms and progressive airflow limitation that collectively contribute to significant morbidity, mortality, and healthcare expenditure worldwide (1, 2). The pathological hallmarks of COPD encompass emphysema, characterized by the irreversible destruction of alveolar walls, and chronic bronchitis, involving inflammation and remodeling of the small airways (3, 4). Long-term exposure to noxious particles and gases, most notably cigarette smoke and environmental pollutants, is the primary etiological factor. These insults initiate a complex and self-perpetuating inflammatory cascade within the lungs, drawing in and dysregulating both innate and adaptive immune cells (5, 6).

**Figure 1:**
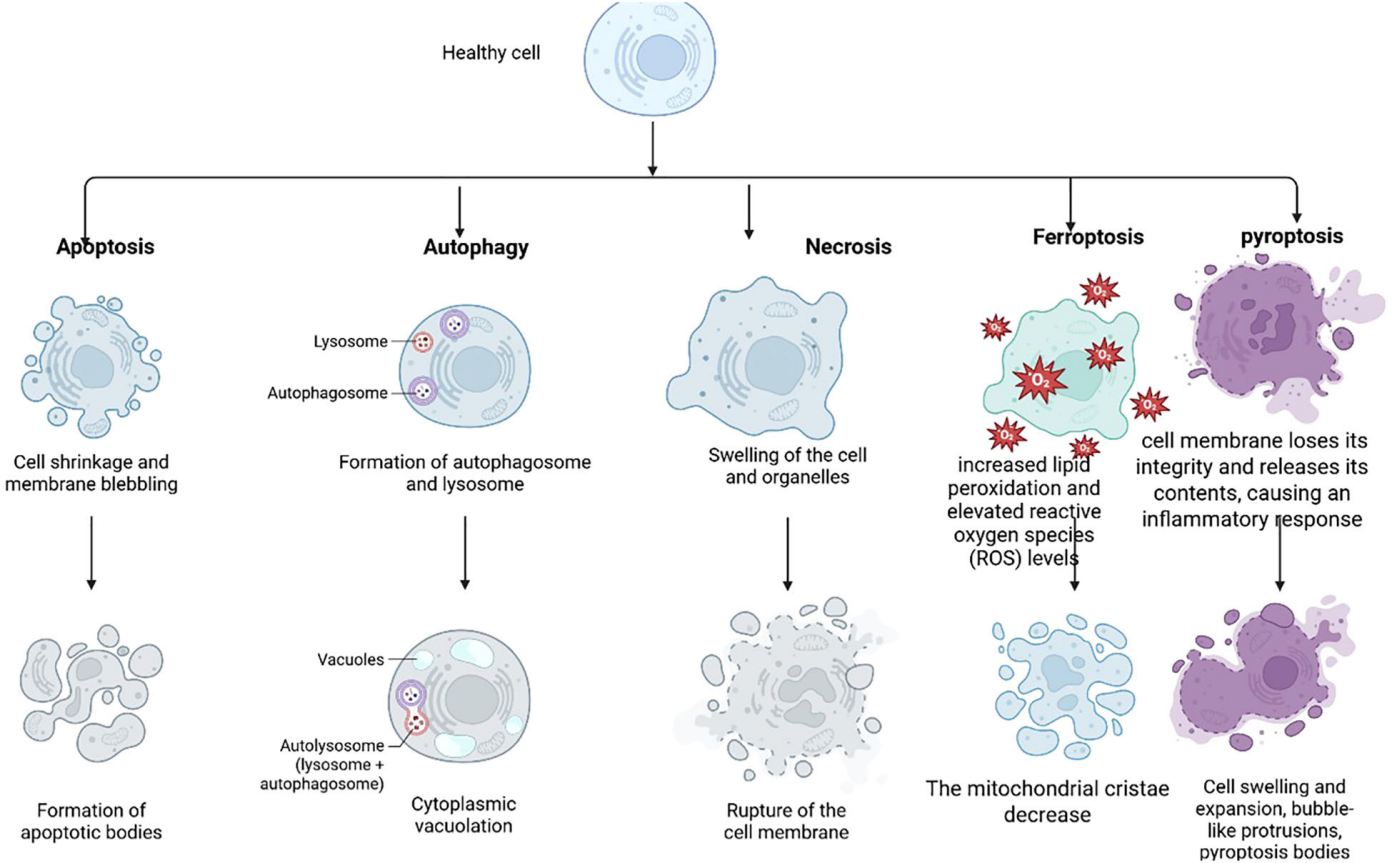
Overview of various cell death modalities and their key characteristics. provides a systematic overview of major regulated cell death pathways—such as apoptosis, necrosis, pyroptosis, ferroptosis, and autophagic cell death—highlighting their key characteristics in a comparative manner. The figure clearly illustrates the distinguishing features of each cell death modality across several dimensions, including: morphological changes (e.g., cell membrane integrity, nuclear condensation, formation of apoptotic bodies); molecular mechanisms (e.g., core signaling pathways, key executioner molecules/proteins, caspase dependence); inducing factors (e.g., types of cellular stress, pathogen infection, signaling molecules); and biological functions (e.g., maintenance of homeostasis, pathogen defense, implications in disease pathogenesis). This visualization serves as an intuitive and valuable reference for understanding the fundamental distinctions, interconnections, and physiological or pathological significance of various cell death pathways.

This chronic, dysregulated immune response creates a vicious cycle of tissue damage and failed repair. Crucially, the mode of cell death within this inflammatory microenvironment is now recognized as one of the key determinants of disease pathology. While apoptosis is generally non-inflammatory, other forms of regulated cell death are potent drivers of inflammation (**Figure 1**). Among these, pyroptosis has emerged as a pivotal mechanism (7). Pyroptosis is a lytic, pro-inflammatory form of programmed cell death canonically triggered by inflammasome complexes that activate caspase-1, which in turn cleaves gasdermin D (GSDMD). The N-terminal fragment of GSDMD oligomerizes to form pores in the plasma membrane, facilitating the release of potent pro-inflammatory cytokines of the IL-1 family and culminating in cell rupture (8). The relevance of pyroptosis to COPD is increasingly evident. Inflammasome components and active GSDMD fragments are upregulated in the lungs of COPD patients and correlate with disease severity (9–11). Cigarette smoke, the principal COPD risk factor, can activate the NLRP3 inflammasome in airway epithelial cells and macrophages, directly linking environmental exposure to this inflammatory cell death pathway (12). The ensuing pyroptosis not only damages the structural integrity of the airway and alveolar epithelium but also creates a feed-forward loop of inflammation (13).

This review synthesizes current evidence from primary research to elaborate on the molecular mechanisms of pyroptosis and its specific contribution to immune dysregulation in COPD. We will delve into how pyroptosis is activated in different lung cell types, how it disrupts immune homeostasis across diverse COPD endotypes, and how it contributes to key pathological features. Finally, we will critically evaluate the burgeoning field of therapeutic interventions aimed at modulating pyroptosis, discussing both the promising avenues and the potential challenges in translating these strategies into clinical benefits for COPD patients.

## 2 Molecular mechanisms of pyroptosis in COPD

### 2.1 Gasdermin-mediated pyroptosis execution

**Figure 2:**
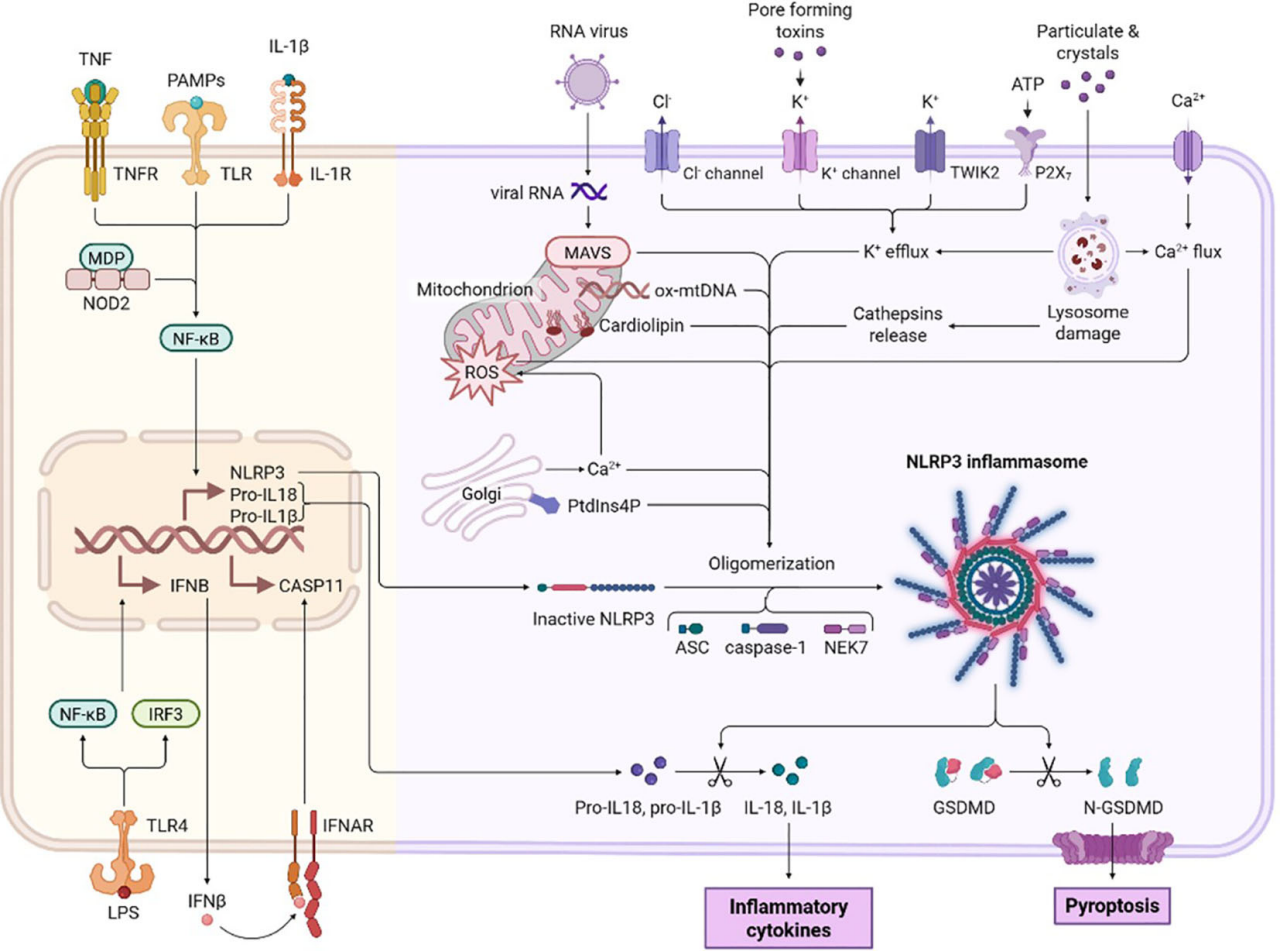
Illustrates that the canonical pathway is the main mechanism behind pyroptosis. This figure demonstrates that the canonical pathway serves as the primary mechanism underlying pyroptosis—a pro-inflammatory programmed cell death process. It visually highlights the core role of the canonical pathway in driving pyroptosis, potentially by illustrating key molecular components (e.g., inflammasomes, caspase family proteins such as caspase-1/4/5/11, and gasdermin D) and their sequential interactions or activation cascades involved in the pathway.

The gasdermin protein family represents the fundamental executioners of pyroptosis, with six identified members in humans (GSDMA-GSDMF) exhibiting distinct but overlapping functions in programmed cell death (14). The functional consequences of GSDMD pore formation extend beyond simple cytokine release. Recent cryo-EM studies have elucidated that these pores exhibit remarkable selectivity, preferentially permitting the passage of folded cytokines like IL-1β, suggesting a sophisticated mechanism for regulated inflammatory signaling (**Figure 2**) (15). In COPD contexts, cigarette smoke exposure significantly upregulates GSDMD expression in human airway epithelial cells (12). GSDMD has emerged as the primary mediator of pyroptosis across multiple cell types relevant to COPD pathogenesis (16). Furthermore, emerging evidence suggests crosstalk between different regulated cell death pathways. A landmark study published in 2025 identified Ferroptosis-Related Genes (FRGs), MDM2 and CDKN1A, as pivotal biomarkers in COPD, correlating with neutrophil-associated inflammation (17). This indicates that co-regulation of pyroptosis and ferroptosis may synergistically contribute to COPD pathogenesis, opening new avenues for combination therapy.

Structural analyses reveal that gasdermin proteins share a conserved architecture featuring an N-terminal pore-forming domain (p30) and a C-terminal autoinhibitory domain (p20) connected by a flexible linker region (18). Under physiological conditions, intramolecular interactions between these domains maintain GSDMD in an autoinhibited state, preventing spontaneous pore formation and uncontrolled cell death. Proteolytic cleavage at specific sites-particularly aspartate 275 (D275) in human GSDMD-liberates the N-terminal fragment from autoinhibition, enabling its oligomerization and insertion into cellular membranes (19). This process generates stable pores approximately 10–15 nanometers in diameter that disrupt plasma membrane integrity and facilitate the release of mature inflammatory cytokines, including IL-1β and IL-18. Recent cryo-electron microscopy studies have elucidated the molecular architecture of GSDMD pores, revealing 26–28 protomer assemblies that form non-selective channels in lipid bilayers (18). These structural insights demonstrate how conformational changes expose hydrophobic regions critical for membrane insertion and subsequent pore stabilization.

The functional consequences of GSDMD pore formation extend beyond simple cytokine release. These pores exhibit remarkable selectivity, preferentially permitting the passage of folded cytokines over larger cellular proteins, suggesting a sophisticated mechanism for regulated inflammatory signaling rather than indiscriminate cellular content release (19). This selective permeability maintains cellular integrity temporarily while ensuring efficient cytokine secretion, creating a controlled inflammatory response. In COPD contexts, cigarette smoke exposure significantly upregulates GSDMD expression in human airway epithelial cells, potentially enhancing cellular susceptibility to pyroptotic cell death (16). Furthermore, oxidative stress conditions characteristic of the COPD microenvironment promotes GSDMD oligomerization through disulfide bond formation, establishing a feed-forward loop of inflammatory signaling (20, 21).

### 2.2 canonical inflammasome-mediated pyroptosis

The canonical pyroptosis pathway represents the most extensively characterized mechanism of inflammatory cell death in COPD. This pathway initiates with pattern recognition receptors (PRRs) detecting either pathogen-associated molecular patterns (PAMPs) or damage-associated molecular patterns (DAMPs) (22). In the specific context of COPD, relevant DAMPs include various cigarette smoke components, extracellular ATP released from damaged cells, uric acid crystals formed during cellular stress, and mitochondrial DNA liberated through oxidative damage (12). These danger signals activate sophisticated molecular platforms known as inflammasomes, which serve as critical signaling hubs in COPD pathogenesis.

Several inflammasome complexes demonstrate particular relevance to COPD progression. The NLRP3 inflammasome responds to diverse stimuli including reactive oxygen species (ROS), lysosomal disruption, and ionic flux disturbances, all commonly observed in cigarette smoke-exposed airways (23). The AIM2 inflammasome recognizes cytosolic DNA from both pathogens and damaged host mitochondria, while the NLRC4 inflammasome specifically detects bacterial flagellin and type III secretion system components, making it particularly relevant during infectious exacerbations (24).

Inflammasome activation triggers a meticulously coordinated assembly process. The adapter protein ASC (apoptosis-associated speck-like protein containing a CARD) forms prion-like filaments that aggregate into distinctive cytoplasmic specks, serving as platforms for pro-caspase-1 recruitment and activation (22). This process exemplifies the remarkable specificity of innate immune signaling, ensuring appropriate cellular responses to distinct danger signals. Active caspase-1 subsequently cleaves GSDMD at the critical D275 site, generating the pore-forming N-terminal fragment while simultaneously processing pro-IL-1β and pro-IL-18 into their biologically active forms (16, 25). These mature cytokines are then released through GSDMD pores in a controlled manner, initiating and amplifying local inflammatory responses.

The clinical relevance of canonical pyroptosis in COPD is substantiated by multiple lines of evidence. Bronchoalveolar lavage fluid from COPD patients contains elevated levels of active caspase-1 and IL-1β compared to healthy controls (8, 26). Genetic studies have identified polymorphisms in NLRP3 and IL-1β genes that associate with COPD susceptibility and severity (8). Furthermore, preclinical models demonstrate that NLRP3 deficiency attenuates cigarette smoke-induced emphysema and airway inflammation, highlighting the therapeutic potential of targeting this pathway (27).

### 2.3 Non-canonical pyroptosis pathway

The non-canonical pyroptosis pathway operates independently of inflammasome complexes and caspase-1 activation, representing an alternative mechanism of inflammatory cell death particularly relevant during infectious exacerbations of COPD. This pathway is mediated by human caspases-4 and -5 (or their murine ortholog caspase-11), which function as both sensors and effectors of intracellular lipopolysaccharide (LPS) (28, 29). Structural studies have revealed that these inflammatory caspases contain characteristic CARD domains that directly bind the lipid A moiety of LPS, triggering caspase autoprocessing and subsequent activation.

Upon activation, caspases-4/5/11 directly cleave GSDMD at the same site targeted by caspase-1, generating the pore-forming N-terminal fragment and initiating pyroptotic cell death (12, 29). However, unlike canonical pyroptosis, the non-canonical pathway does not directly process pro-IL-1β and pro-IL-18 into their mature forms. Instead, it induces cytokine maturation and release through an elegant secondary mechanism involving potassium efflux-mediated activation of the NLRP3 inflammasome (30). This creates sophisticated amplification loops between canonical and non-canonical pathways during bacterial infections, potentially explaining the exaggerated inflammatory responses observed during infectious exacerbations of COPD.

The clinical significance of non-canonical pyroptosis in COPD is particularly evident during bacterial exacerbations. Gram-negative bacteria such as Haemophilus influenzae and Pseudomonas aeruginosa, frequently isolated from COPD airways during exacerbations, release substantial amounts of LPS that can activate this pathway (31). Importantly, impaired mucociliary clearance and defective phagocytosis in COPD patients may facilitate bacterial persistence and increased intracellular LPS exposure, further enhancing non-canonical pyroptosis activation (32). Recent evidence suggests that cigarette smoke exposure potentiates non-canonical pyroptosis by upregulating caspase-4 expression in airway epithelial cells and impairing autophagy-mediated LPS clearance (12, 33).

### 2.4 Granzyme mediated pyroptosis

Beyond inflammasomes, granzyme-mediated pyroptosis represents an important link between adaptive immunity and inflammatory cell death (34, 35). Cytotoxic lymphocytes, including CD8+ T cells and natural killer (NK) cells, eliminate target cells by releasing perforin and granzyme serine proteases (36, 37). Perforin facilitates granzyme entry into the target cell, where they typically trigger apoptosis (38, 39). However, a recently discovered pathway involves granzyme A directly cleaving GSDMB at a unique site, liberating its pore-forming domain and inducing pyroptosis independently of caspase activation (34). In COPD, CD8^+^ T cells infiltrating the lungs express elevated granzyme levels, potentially contributing to epithelial damage through this pathway (40). Viral infections that trigger COPD exacerbations strongly activate cytotoxic lymphocytes, potentially enhancing granzyme-mediated pyroptosis of infected epithelial cells (35). A comparative summary of the molecular initiators, key effectors, COPD context, and therapeutic implications of these pyroptosis pathways is provided in **Table 1**.

**Table 1:** Comparative analysis of pyroptosis pathways in COPD pathogenesis.

Pathway	Molecular initiators	Key effector molecules	COPD context	Therapeutic implications	References
Canonical	NLRP3, AIM2, NLRC4 inflammasomes	Caspase-1, GSDMD, ASC	Chronic smoke exposure, Sterile inflammation, Oxidative stress	NLRP3 inhibitors, Caspase-1 inhibitors, ASC-targeting strategies	(41)
Non-canonical	Intracellular LPS from Gram-negative bacteria	Caspase-4/5/11, GSDMD	Bacterial exacerbations, Impaired bacterial clearance	Caspase-4/5 inhibitors, LPS-neutralizing approaches, Enhanced bacterial clearance	(42)
Granzyme-mediated	Cytotoxic lymphocytes (CD8+ T cells, NK cells)	Granzyme A, GSDMB	Viral exacerbations, Adaptive immune activation, Chronic inflammation	GSDMB modulation, Lymphocyte regulation, Antiviral strategies	(16)

## 3 Cell-type-specific pyroptosis in COPD

### 3.1 Airway epithelial cells: the first line of defense and its collapse

**Figure 3:**
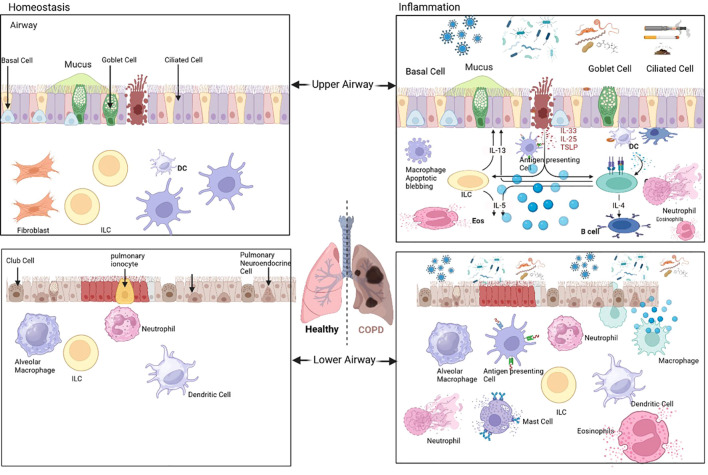
Key cell types in the lungs during chronic obstructive pulmonary disease (COPD). The left panel depicts the upper and lower airway cell types that contribute to pulmonary homeostasis, while the right panel illustrates the cellular alterations associated with COPD. Images were created using BioRender.com. The left panel identifies the cell types involved in maintaining the homeostasis of the lung, encompassing both the upper and lower airways; these cells play pivotal roles in preserving normal pulmonary structure and physiological functions. The right panel delineates the alterations in lung cell types (e.g., phenotypic transition, abnormal proliferation, or functional dysregulation) that occur during the pathogenesis and progression of COPD, thereby reflecting the cellular-level pathological features of the lung tissue under disease conditions.

The airway epithelium represents a sophisticated barrier system that undergoes profound pyroptotic activation in COPD, serving as both a victim and amplifier of inflammatory signaling (12). Primary human bronchial epithelial cells isolated from COPD patients demonstrate significantly enhanced NLRP3 expression and caspase-1 activation following cigarette smoke exposure, with response magnitudes correlating with disease severity (43). Single-cell RNA sequencing analyses have identified distinct epithelial subpopulations with varying pyroptosis susceptibility, including a specialized GSDMD-high cluster that expands during disease progression and may contribute to COPD heterogeneity (**Figure 3**) (44, 45).

The molecular mechanisms underlying epithelial pyroptosis involve multi-faceted pathways. Cigarette smoke induces comprehensive mitochondrial dysfunction through complex I inhibition and cytochrome c release, generating substantial reactive oxygen species (ROS) that activate the NLRP3 inflammasome via thioredoxin-interacting protein (TXNIP) dissociation and potassium efflux (46, 47). Additionally, smoke components including acrolein and cadmium directly damage lysosomal membranes, releasing cathepsins B and L that promote inflammasome assembly through dual mechanisms: direct NLRP3 activation and mitochondrial DNA degradation (48). During infectious exacerbations, viral pathogens such as rhinovirus and influenza engage multiple pattern recognition receptors (PRRs) including RIG-I and TLR3, while bacterial components activate NLRP3 and AIM2 inflammasomes, creating synergistic amplification of pyroptotic responses (49, 50).

The functional consequences extend far beyond simple cellular demise. GSDMD pores facilitate selective release of IL-1β and IL-18 while maintaining membrane integrity for several hours, creating sustained inflammatory signaling. This process recruits and activates neutrophils and macrophages through chemotactic gradient establishment (15). Critically, epithelial barrier disruption increases infection susceptibility by reducing tight junction protein expression and mucociliary clearance, establishing self-perpetuating inflammation-injury cycles (51). Recent evidence suggests that persistent epithelial pyroptosis may also drive abnormal repair mechanisms through fibroblast growth factor (FGF) and transforming growth factor-β (TGF-β) pathway dysregulation, contributing to small airway fibrosis (52).

### 3.2 Alveolar macrophages: orchestrators of inflammation

Alveolar macrophages undergo fundamental phenotypic and functional reprogramming in COPD, transitioning from homeostatic regulators to primary drivers of pyroptotic inflammation (53). These sentinel immune cells demonstrate a marked shift toward pro-inflammatory M1 polarization with enhanced inflammasome component expression and pyroptosis susceptibility (54). Single-cell analyses reveal distinct macrophage subpopulations in COPD lungs, including a novel NLRP3-high subset that not only correlates with emphysema severity (45) but also aligns with the ‘Emphysema-Dominant’ phenotype described in Section 4.2, providing a cellular basis for this clinical presentation.

Cigarette smoke exposure initiates macrophage pyroptosis priming through multiple synchronized mechanisms. NF-κB activation upregulates NLRP3 and pro-IL-1β expression while simultaneously downregulating anti-inflammatory mediators (55). Mitochondrial dysfunction induces cardiolipin translocation to outer membranes, creating direct NLRP3 activation platforms (56, 57). Impaired autophagic flux reduces clearance of damaged organelles and protein aggregates, accumulating inflammasome-activating materials. Additionally, oxidative stress conditions promote sustained inflammasome activation through TXNIP dissociation and histone modification-mediated epigenetic reprogramming.

A critical aspect of macrophage dysfunction in COPD involves defective efferocytosis-the clearance of apoptotic cells-which amplifies pyroptotic responses by allowing secondary necrosis and damage-associated molecular pattern (DAMP) release. This creates a vicious cycle where impaired clearance capacity exacerbates inflammatory burden. Macrophage pyroptosis directly contributes to emphysema pathogenesis through IL-1β-stimulated matrix metalloproteinase (MMP-9 and MMP-12) production and fibroblast activation, driving extracellular matrix degradation and abnormal tissue remodeling (58).

### 3.3 Neutrophils: amplifiers of inflammatory cascades

Neutrophil infiltration represents a cardinal feature of COPD pathology, and emerging evidence positions pyroptosis as a crucial regulator of neutrophil function and survival dynamics (59). While neutrophils primarily utilize NETosis for pathogen defense, they undergo significant pyroptosis during bacterial infections through caspase-4/5 activation pathways (19, 60, 61). Recent single-cell proteomic analyses reveal that neutrophils from COPD patients exhibit enhanced expression of pyroptosis-related proteins, including elevated caspase-4 levels that correlate with exacerbation frequency (62, 63). The activation of non-canonical pyroptosis in neutrophils during bacterial infections positions them as key drivers in the “Frequent Exacerbator Phenotype”.

Multiple factors converge to promote neutrophil pyroptosis in the COPD microenvironment. Bacterial infections common during exacerbations activate caspase-4/5 through intracellular LPS detection, while cigarette smoke induces mitochondrial DNA release that activates the AIM2 inflammasome (64). The characteristic protease-antiprotease imbalance may process gasdermins through non-canonical mechanisms, with neutrophil elastase demonstrating direct GSDMD cleavage capacity under specific conditions. Additionally, oxidative stress promotes disulfide bond formation that enhances GSDMD oligomerization efficiency (65).

Neutrophil pyroptosis significantly amplifies inflammatory cascades through IL-1β release and appears to contribute to NET formation through novel mechanistic links (59). GSDMD pores facilitate the extrusion of NET components, creating a hybrid cell death modality that combines features of pyroptosis and NETosis (11, 66). This process may explain the excessive NET formation observed in severe COPD and its association with disease progression. The resulting NETs contain various antimicrobial proteins and DNA webs that damage host tissue and perpetuate inflammation through DAMP-mediated immune activation.

### 3.4 T Lymphocytes: adaptive immune contributions

While traditionally associated with adaptive immunity, T lymphocytes contribute significantly to COPD pathogenesis through both direct pyroptosis and indirect inflammatory amplification (36). Recent single-cell transcriptomic studies demonstrate that CD8^+^ T cells from COPD lungs exhibit increased expression of pyroptosis-related genes, including elevated GSDMD and caspase-1 transcripts that reflect chronic activation states (67). Viral infections commonly trigger pyroptosis in virus-specific T cells through RIG-I-mediated inflammasome activation, potentially explaining the prolonged immunosuppression periods following respiratory infections in COPD patients (68).

The functional consequences of T cell pyroptosis extend across multiple immunological domains. Reduction of virus-specific cytotoxic T cell populations impairs viral clearance capacity, increasing exacerbation severity and duration. Inflammatory cytokine release during pyroptosis amplifies innate immune responses through bystander activation mechanisms (16). Perhaps most intriguingly, neoantigen generation through specific protein cleavage during pyroptosis may trigger autoimmune responses, potentially explaining the autoimmune features observed in some COPD patients (36, 69). Additionally, CD4^+^ T cell pyroptosis may disrupt regulatory T cell function, further amplifying inflammatory cascades (69).

### 3.5 Dendritic cells: bridging innate and adaptive immunity

Dendritic cells (DCs) serve as crucial intermediaries between innate and adaptive immunity, and their pyroptotic regulation significantly influences COPD progression. Multiple DC subsets exhibit distinct pyroptosis susceptibility patterns, with conventional DCs demonstrating enhanced NLRP3 activation compared to plasmacytoid DCs in COPD environments (70). Cigarette smoke exposure induces mitochondrial ROS production in DCs, activating the NLRP3 inflammasome and promoting IL-1β secretion that shapes T cell polarization toward Th1 and Th17 phenotypes (71).

Uric acid crystals produced by damaged epithelial cells further enhance DC pyroptosis through the NLRP3 inflammasome, creating positive feedback loops that sustain inflammation (72). Activated DCs release IL-6 and TNF-α, promoting structural airway changes through fibroblast activation and extracellular matrix remodeling. The airway epithelium regulates DC function through IL-12p40 and chitinase-3-like protein 1 (CHI3L1) secretion, establishing bidirectional communication networks that amplify pyroptotic responses (73).

Age-related changes in DC function significantly impact COPD severity, with aging DCs demonstrating altered antigen-presenting capabilities and enhanced pyroptosis susceptibility through declined autophagy capacity (74). This age-associated vulnerability may explain the accelerated lung function decline in elderly COPD patients and highlights the importance of considering aging mechanisms in therapeutic development.

### 3.6 Myeloid-derived suppressor cells: immunomodulatory paradox

Myeloid-derived suppressor cells (MDSCs) represent a heterogenous population of immature myeloid cells that expand dramatically in COPD and exhibit complex relationships with pyroptosis pathways (75). These cells demonstrate potent immunosuppressive capabilities through arginase-1 production, reactive oxygen species generation, and T cell proliferation inhibition. Interestingly, MDSCs from COPD patients show enhanced pyroptosis resistance through upregulated anti-apoptotic protein expression and improved mitochondrial stability, allowing persistent immunosuppressive function despite inflammatory environments (69).

The accumulation of MDSCs in COPD lungs creates an immunosuppressive microenvironment that paradoxically sustains chronic inflammation through impaired pathogen clearance and alternative macrophage activation. However, MDSC pyroptosis, when it occurs, releases substantial inflammatory mediators that can abruptly amplify tissue damage. This creates a delicate balance where MDSC survival and death decisions significantly influence disease progression trajectories (76).

This comprehensive analysis of cell-type-specific pyroptosis mechanisms reveals the sophisticated cellular networks driving COPD pathogenesis. The primary pathways, triggers, functional consequences, and therapeutic implications for each major lung cell type involved are systematically compared in **Table 2**. The intricate interplay between different cell populations, combined with their unique pyroptosis activation patterns and functional consequences, creates a complex inflammatory landscape that varies across disease stages and individual patients. Understanding these cell-specific mechanisms provides the foundation for developing targeted therapeutic strategies that can precisely modulate pyroptosis in specific cell types while preserving essential immune functions.

**Table 2:** Cell-type-specific pyroptosis characteristics in COPD.

Cell type	Primary pyroptosis pathways	Key molecular triggers	Functional consequences	Therapeutic implications	References
Airway Epithelial Cells	Canonical (NLRP3), Non-canonical	Cigarette smoke, Viral infections, Bacterial components	Barrier dysfunction, Chronic inflammation, Abnormal repair	Inhaled caspase inhibitors, NLRP3 antagonists, Barrier stabilizers	(77)
Alveolar Macrophages	Canonical (NLRP3, AIM2)	Mitochondrial damage, Oxidative stress, DAMPs	MMP production, Fibroblast activation, Efferocytosis impairment	M2 polarization inducers, Autophagy enhancers, Efferocytosis promoters	(78)
Neutrophils	Non-canonical, Granzyme-mediated	Intracellular LPS, Mitochondrial DNA, Protease imbalance	NETosis enhancement, Tissue damage, Inflammation amplification	Caspase-4/5 inhibitors, NETosis modulators, Antioxidants	(79)
T Lymphocytes	Canonical, Granzyme-mediated	Viral infections, Chronic activation, Oxidative stress	Immunosuppression, Autoimmunity initiation, Cytokine storms	Checkpoint modulators, Viral clearance enhancers, Antioxidant approaches	(80)
Dendritic Cells	Canonical (NLRP3)	Uric acid crystals, Mitochondrial ROS, Cellular debris	Altered T cell polarization, Chronic inflammation maintenance	DC reprogramming, TSLP modulation, IL-33 targeting	(81)
MDSCs	Canonical (resistant)	Inflammatory cytokines, Hypoxia, Metabolic stress	Immunosuppression persistence, Inflammation modulation	MDSC differentiation inducers, Metabolic modulators, Selective depletion	(82)

## 4 Pyroptosis in COPD heterogeneity and disease stages

### 4.1 Dynamic pyroptosis regulation across disease continuum

**Figure 4:**
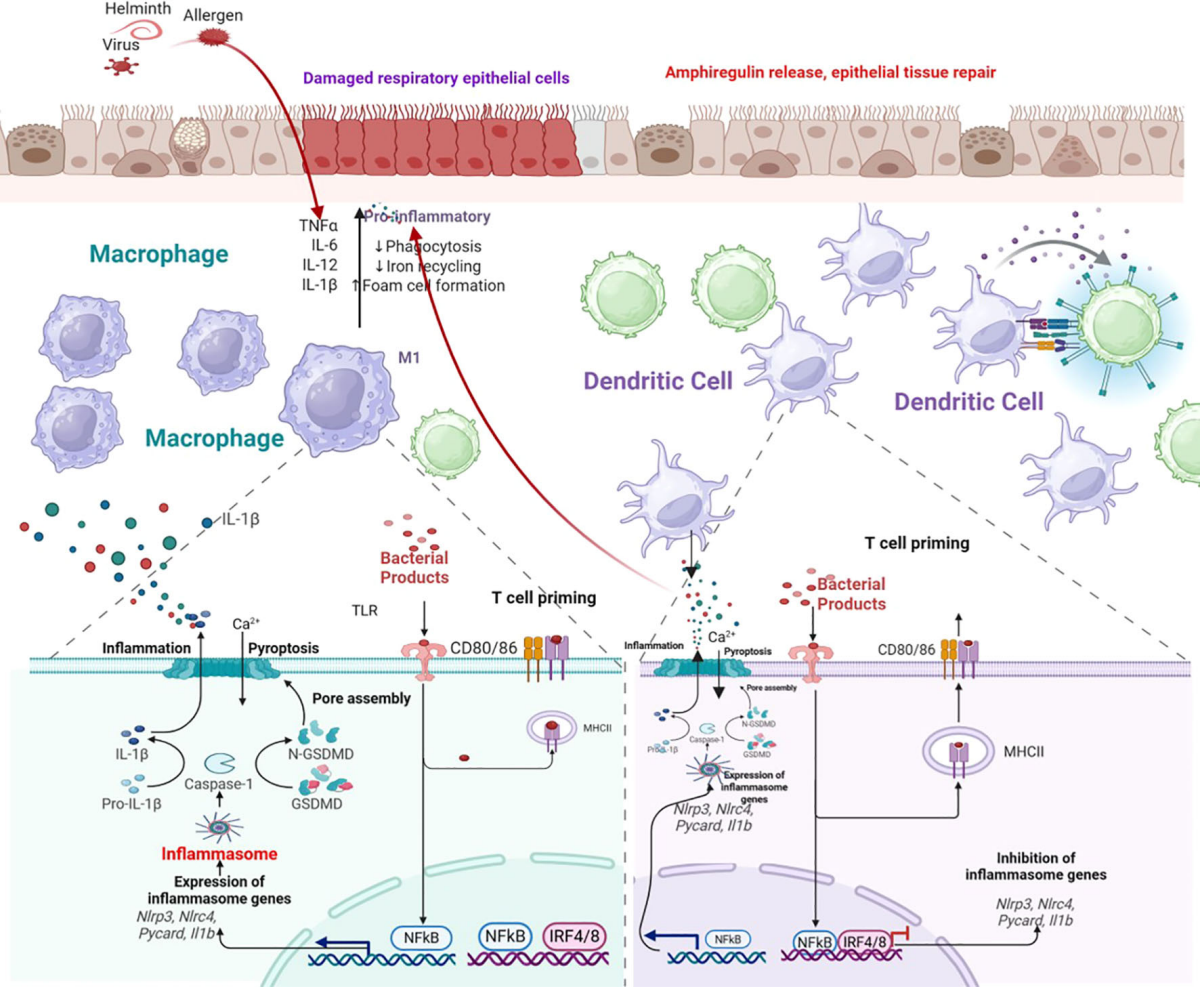
Immune cells promote the damage of respiratory tract epithelium in COPD through pyroptosis. This figure illustrates the critical role of immune cells in driving respiratory tract epithelial injury during chronic obstructive pulmonary disease (COPD) progression, with pyroptosis serving as the key mediating mechanism. It visually depicts the interplay between infiltrating or resident lung immune cells (e.g., macrophages, neutrophils, or T lymphocytes) and airway epithelial cells: activated immune cells release pro-inflammatory factors or directly trigger signaling cascades that induce pyroptosis—a pro-inflammatory programmed cell death—in epithelial cells. Such pyroptotic death disrupts epithelial barrier integrity, impairs mucosal defense functions, and amplifies local inflammatory responses, collectively exacerbating epithelial damage and contributing to the pathological manifestations of COPD (e.g., airway remodeling, mucus hypersecretion). By clarifying this immune cell-pyroptosis-epithelial damage axis, the figure provides valuable insight into the cellular and molecular mechanisms underlying COPD pathogenesis, supporting further exploration of targeted therapeutic strategies.

Pyroptosis activation patterns vary dramatically across the COPD spectrum. In stable COPD, low-grade pyroptosis primarily involves airway epithelial cells and alveolar macrophages, driven by chronic smoke exposure and oxidative stress, maintaining a state of controlled inflammation. During acute exacerbations (AECOPD), pyroptosis intensifies and engages multiple cell populations. Viral pathogens activate RIG-I/MDA5-dependent NLRP3 inflammasomes, while bacterial pathogens activate both canonical and non-canonical pathways (**Figure 4**) (83). This creates a cytokine storm featuring elevated IL-1β and IL-18 that correlates with clinical severity. Biomarker studies show elevated plasma GSDMD fragments during exacerbations that normalize upon recovery (83).

### 4.2 Phenotype-specific pyroptosis signatures

COPD heterogeneity manifests through distinct clinical phenotypes with characteristic pyroptosis signatures. Frequent Exacerbator Phenotype: Shows enhanced NLRP3 inflammasome priming and IL-1β hyperproduction, with peripheral blood mononuclear cells demonstrating exaggerated caspase-1 activation. Genetic polymorphisms in NLRP3 may underlie this phenotype (84). Emphysema-Dominant Phenotype: Exhibits preferential airway epithelial pyroptosis with enhanced alveolar destruction. Type II alveolar epithelial cells show increased AIM2 inflammasome activation driven by mitochondrial DNA release, and circulating GSDMD fragments correlate with emphysema severity (25).

Chronic Bronchitis Phenotype: Features prominent goblet cell metaplasia with distinct pyroptosis regulation, including IL-33-mediated alternative inflammasome activation that stimulates mucin gene expression (85). The dominant pyroptosis pathways, key cellular players, biomarkers, and therapeutic implications across these COPD stages and phenotypes are delineated in **Table 3**.

**Table 3:** Pyroptosis characteristics across COPD stages and phenotypes.

Disease stage/phenotype	Dominant pyroptosis pathways	Key cellular players	Characteristic biomarkers	Therapeutic implications	References
Stable COPD	Canonical (NLRP3)	Airway epithelial cells, Alveolar macrophages	Moderate IL-18, GSDMD fragments	Maintenance anti-inflammatories, Oxidative stress reduction	(13)
AECOPD	Canonical + Non-canonical	Epithelial cells, Macrophages, Neutrophils	High IL-1β, IL-18, GSDMD fragments	Broad-spectrum caspase inhibitors, Pathogen-directed therapy	(86)
Frequent Exacerbator	Enhanced NLRP3	Monocytes, Airway epithelium	Elevated caspase-1 activity, IL-1β hyperproduction	NLRP3 inhibitors, IL-1 receptor antagonists	(84)
Emphysema-Dominant	AIM2-mediated	Type II alveolar cells, Macrophages	Mitochondrial DNA, GSDMD-CTF	AIM2 inhibitors, Mitochondrial stabilizers	(87)
Chronic Bronchitis	IL-33-mediated	Goblet cells, Airway epithelium	IL-33, TSLP, Mucin proteins	IL-33 pathway blockers, Mcoregulators	(88)

### 4.3 Molecular determinants of pyroptosis heterogeneity

Genetic polymorphisms significantly contribute to pyroptosis heterogeneity in COPD, with genome-wide association studies identifying specific variants in NLRP3, CARD8, and IL-1β genes that associate with disease susceptibility and progression trajectories (89). The NLRP3 Q705K polymorphism enhances inflammasome assembly stability, while CARD8 variants regulate inflammasome inhibition efficiency, collectively modulating individual pyroptosis thresholds (90). Additionally, GSDMD polymorphisms influence pore formation kinetics, creating variability in inflammatory output from similar levels of caspase activation.

Epigenetic modifications induced by environmental exposures create lasting pyroptosis programming that varies across COPD subtypes. DNA methylation analyses reveal distinct patterns in inflammation- and pyroptosis-related genes (91). Beyond the pathways and cellular players, the heterogeneity of pyroptosis in COPD is also shaped by systemic and microenvironmental factors. The role of autoimmune components is gaining recognition, with studies demonstrating the presence of tertiary lymphoid structures and autoantibodies in COPD lungs, which may perpetuate pyroptosis and inflammation even after smoking cessation (69). Additionally, dysbiosis of the airway microbiome creates a pro-inflammatory milieu that can prime inflammasons. A 2024 study confirmed that alterations in airway microbiota composition are significantly associated with immune activation and COPD severity, suggesting that microbial-derived signals may be a key variable in determining an individual’s pyroptosis activation threshold and subsequent disease phenotype.

### 4.5 Future research directions

Several critical knowledge gaps require addressing to fully leverage pyroptosis heterogeneity for therapeutic benefit. Longitudinal studies tracking pyroptosis biomarker evolution across disease transitions could identify critical intervention points for preventing progression. Advanced imaging techniques capable of visualizing pyroptosis activation in real-time would enable precise assessment of therapeutic responses. Furthermore, understanding how pyroptosis interfaces with other cell death pathways in different COPD contexts may reveal novel regulatory nodes for therapeutic manipulation.

The development of phenotype-specific animal models that recapitulate human pyroptosis heterogeneity represents another crucial direction. Such models would facilitate preclinical testing of targeted therapies and provide insights into the molecular mechanisms driving phenotype divergence. Additionally, exploring the interaction between pyroptosis and the lung microbiome across different COPD phenotypes may reveal novel pathogen-host interactions that influence disease course and treatment responses. This comprehensive understanding of pyroptosis heterogeneity across COPD stages and phenotypes provides the foundation for developing precisely targeted therapeutic strategies that address specific molecular mechanisms in defined patient subgroups, moving beyond the current one-size-fits-all approach to COPD management.

## 5 Therapeutic implications and clinical translation

### 5.1 Strategic targeting of inflammasome complexes

Therapeutically targeting inflammasome complexes represents a promising approach for COPD management, given their upstream position in pyroptosis initiation. NLRP3 inhibition has demonstrated particular promise, with several candidates advancing through preclinical and clinical development. MCC950, a potent and specific NLRP3 inhibitor, significantly reduces inflammation and emphysema progression in cigarette smoke-exposed murine models by blocking NLRP3 ATPase activity and subsequent inflammasome assembly (92). However, despite promising efficacy, clinical development faced challenges due to unforeseen toxicity profiles, highlighting the importance of comprehensive safety assessment in pyroptosis-targeted therapies.

OLT1177, an oral β-sulfonyl nitrile compound, exhibits favorable safety profiles in early-phase clinical trials for other inflammatory conditions while effectively inhibiting NLRP3-mediated IL-1β production (93). Its potential application in COPD requires dedicated clinical evaluation, particularly regarding optimal dosing regimens and long-term safety in elderly populations with comorbidities. Tranilast, an FDA-approved antiallergic medication recently identified as an NLRP3 inhibitor, offers immediate repurposing opportunities for COPD management, though its relatively weak inhibitory potency may limit therapeutic efficacy in advanced disease stages (94).

Clinical implementation of inflammasome inhibitors necessitates careful consideration of host defense preservation. Intermittent administration during high-risk periods or exacerbation seasons may balance anti-inflammatory efficacy with infection risk mitigation. Inhaled delivery approaches could maximize lung targeting while minimizing systemic exposure, potentially reducing off-target effects. Biomarker-guided patient selection, focusing on individuals with elevated NLRP3 activity or specific genetic polymorphisms, may optimize therapeutic responses while minimizing treatment exposure in unlikely responders.

### 5.2 Gasdermin-targeted intervention strategies

Direct targeting of gasdermin proteins offers a complementary approach downstream of inflammasome activation, potentially providing broader anti-pyroptotic effects across multiple activation pathways. Necrosulfonamide effectively blocks GSDMD pore formation by covalently modifying cysteine 191 (95). However, its clinical translation faces significant hurdles, including poor solubility and potential off-target effects due to its non-specific reactivity with other cysteine-containing proteins. Innovative delivery systems, such as nanoparticle encapsulation, are being explored to overcome these limitations.

Dimethyl fumarate, an FDA-approved medication for multiple sclerosis, recently demonstrated GSDMD inhibitory activity through covalent modification, presenting immediate repurposing potential for COPD management (20). Its established safety profile and oral administration route facilitate rapid clinical evaluation, though COPD-specific dosing regimens and efficacy endpoints need determination. Disulfiram, an alcohol-aversion drug, inhibits GSDMD pore formation and shows protective effects in sepsis models, though its relatively narrow therapeutic window and neurological side effects at higher doses may limit clinical utility in COPD populations (96).

Gasdermin inhibition presents unique challenges, including potential intracellular cytokine accumulation due to blocked release mechanisms and compensatory activation of alternative gasdermin family members. Combination approaches targeting multiple gasdermins or implementing sequential inhibition strategies may overcome these limitations. Additionally, cell-type-specific delivery systems could enhance therapeutic precision while minimizing systemic effects.

### 5.3 Caspase inhibition strategies

Caspase inhibition represents another strategic approach, targeting key executioners common to multiple pyroptosis pathways. VX-765, an orally available caspase-1 inhibitor, demonstrated acceptable safety profiles in phase II epilepsy trials and shows promise for reducing IL-1β-mediated inflammation in COPD (97). However, COPD-specific efficacy trials are necessary to establish optimal dosing, treatment duration, and patient selection criteria.

Caspase-4/5 inhibitors currently in early development stages offer potential for specifically targeting infection-driven pyroptosis during COPD exacerbations. Their selective inhibition may preserve canonical pathway functions while controlling excessive non-canonical activation during bacterial challenges (98). However, the structural similarity between inflammatory caspases presents challenges for achieving absolute specificity, requiring sophisticated molecular design approaches.

Pan-caspase inhibitors like emricasan demonstrate broad anti-pyroptotic effects but raise concerns regarding essential apoptotic pathway disruption and potential oncogenic risk with long-term administration (98). The development of caspase-specific inhibitors with limited tissue distribution or inhaled formulations may mitigate these concerns while maintaining therapeutic efficacy.

### 5.4 Integrated and personalized therapeutic approaches

Given COPD’s complexity and heterogeneity, integrated therapeutic strategies likely offer superior efficacy compared to single-pathway interventions. Combining pyroptosis inhibitors with established COPD therapies presents synergistic opportunities. For instance, VX-765 combined with inhaled corticosteroids may enhance anti-inflammatory effects while enabling corticosteroid dose reduction, potentially mitigating steroid-related side effects (47). Similarly, gasdermin inhibitors combined with long-acting bronchodilators may address both inflammatory and bronchoconstrictive components simultaneously.

Coordinated regulation of different cell death pathways represents another integrative approach. Simultaneously modulating pyroptosis and apoptosis through carefully balanced inhibitor combinations may better preserve tissue homeostasis than single-pathway targeting. This approach requires sophisticated understanding of cell death pathway interactions in specific COPD contexts and careful dosing to avoid excessive immunosuppression. Personalized approaches based on individual pyroptosis activation patterns offer promising directions for precision medicine in COPD. Biomarker-driven patient stratification using GSDMD cleavage products, inflammasome activity assays, or genetic polymorphism profiles could optimize therapeutic matching. Phenotype-specific therapy selection may include NLRP3 inhibitors for frequent exacerbators, AIM2 inhibitors for emphysema-dominant patients, and IL-33 pathway modulators for chronic bronchitis phenotypes. A comprehensive assessment of key pyroptosis-targeting therapeutic candidates, including their mechanisms, development stages, and associated challenges, is presented in **Table 4**.

**Table 4:** Comprehensive assessment of pyroptosis-targeting therapeutic candidates.

Therapeutic target	Candidate agents	Mechanism of action	Development stage	Advantages	Limitations and challenges	References
NLRP3 Inflammasome	MCC950, OLT1177, Tranilast, Dapansutrile	Direct NLRP3 inhibition, ATPase blockade	Preclinical to Phase II	Upstream targeting, broad anti-inflammatory effects	Host defense impairment, toxicity concerns, compensatory pathway activation	(99)
Alternative Inflammasomes	AIM2 inhibitors (developmental), NLRC4 antagonists	DNA sensing inhibition, NAIP interaction blockade	Early preclinical	Phenotype-specific targeting, infection context specificity	Limited candidate availability, narrow therapeutic windows	(100)
GSDMD	Necrosulfonamide, Dimethyl fumarate, Disulfiram	Pore formation blockade, cysteine modification	Preclinical to FDA-approved	Downstream effector targeting, multi-pathway coverage	Intracellular cytokine accumulation, gasdermin compensation, formulation challenges	(101)
Inflammatory Caspases	VX-765, Caspase-4/5 inhibitors, Emricasan	Catalytic site inhibition, pro-domain interaction	Phase II to preclinical	Pathway convergence targeting, well-characterized targets	Apoptosis pathway disruption, infection risk, specificity limitations	(47)
Combination Approaches	Inhaled corticosteroids + caspase inhibitors, Bronchodilators + gasdermin inhibitors	Multi-pathway modulation, synergistic anti-inflammatory effects	Concept stage to early clinical	Enhanced efficacy, side effect reduction, personalized application	Complex dosing regimens, drug interaction concerns, regulatory challenges	(102)

### 5.5 Clinical translation challenges and future directions

**Figure 5:**
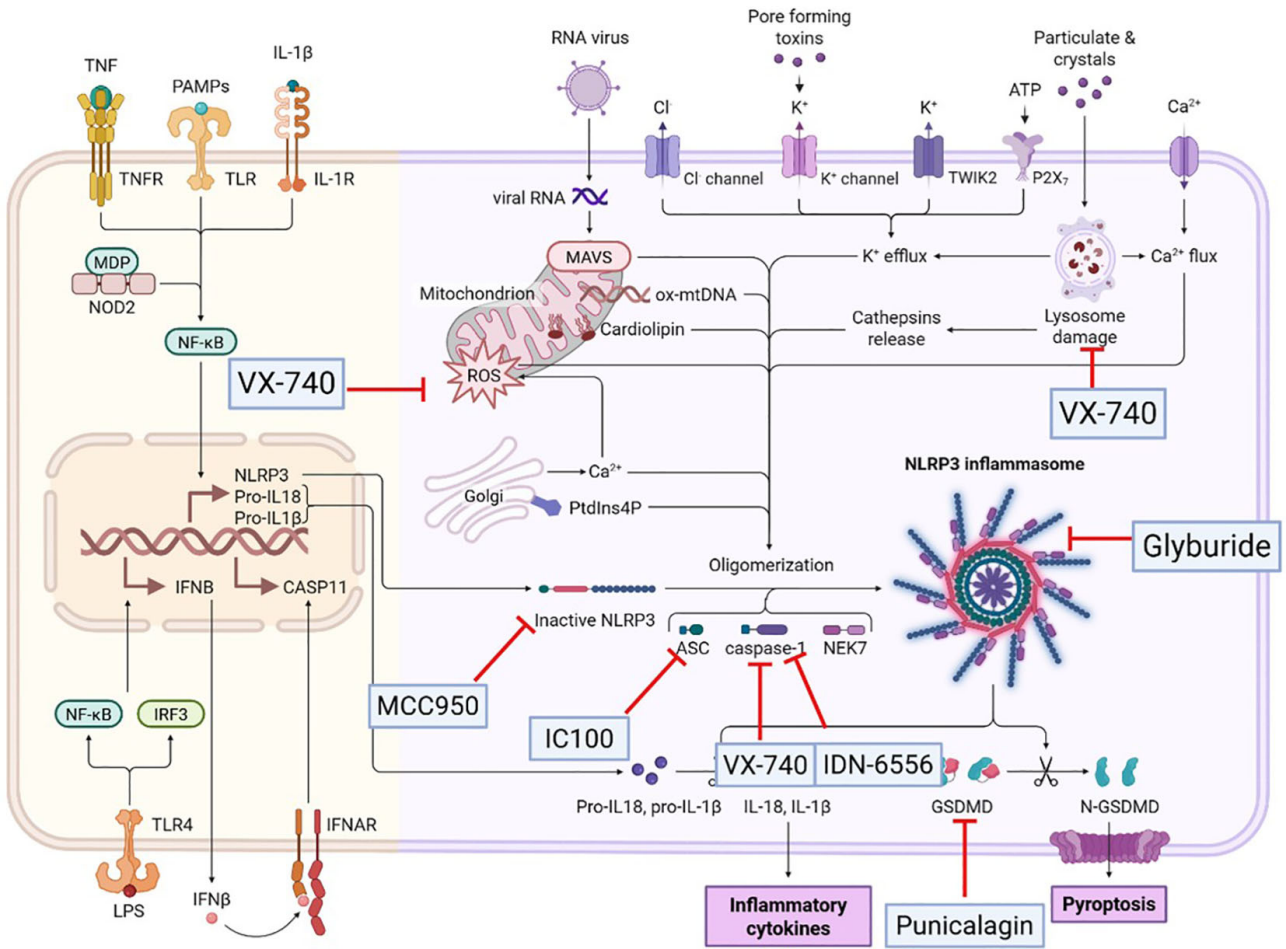
Potential strategies targeting pyroptosis for the treatment of COPD. This figure systematically outlines promising therapeutic strategies aimed at modulating pyroptosis to treat COPD, a progressive respiratory disorder characterized by persistent airway inflammation and structural damage. It visually presents multiple targeted approaches that interfere with key nodes of the pyroptotic pathway, including (but not limited to) inhibition of inflammasome assembly (e.g., targeting NLRP3), suppression of caspase activation (e.g., caspases-1/4/5/11), and regulation of gasdermin D (GSDMD) cleavage or pore formation—core molecular events driving pyroptotic cell death. Additionally, the figure may highlight strategies targeting cell-specific pyroptosis (e.g., in lung immune cells or epithelial cells) to mitigate excessive inflammation while preserving normal cellular function. By delineating these candidate interventions, the figure offers a clear, actionable framework for translating pyroptosis-focused research into novel COPD therapies, underscoring the potential of targeting this pro-inflammatory cell death pathway to alleviate disease progression.

The translation of pyroptosis-targeting therapies from preclinical models to clinical practice faces several significant challenges (**Figure 5**). Optimal timing and duration of intervention require careful consideration, as pyroptosis plays essential physiological roles in host defense and cellular homeostasis. Biomarker-driven intermittent dosing or exacerbation-focused treatment strategies may balance therapeutic benefits with risk mitigation. Drug delivery optimization represents another critical challenge. Inhaled formulations using nanoparticle carriers or liposomal systems could enhance lung-specific targeting while minimizing systemic exposure. Advanced delivery technologies enabling selective cell-type targeting may further improve therapeutic precision, particularly for strategies targeting immune cells versus structural cells.

Long-term safety assessment remains paramount, especially regarding cancer surveillance, infection susceptibility, and tissue repair capacity. Comprehensive post-marketing surveillance and registry studies will be essential for detecting rare or delayed adverse effects not identified in limited-duration clinical trials. The translation of pyroptosis-targeting therapies faces the paramount challenge of preserving essential host defense against pathogens. Complete suppression of pyroptosis may compromise bacterial clearance, potentially increasing infection risk. Strategic approaches, such as intermittent dosing during high-risk periods or the development of inhaled, lung-specific formulations, are crucial to mitigate this risk. Inhaled formulations using nanoparticle carriers could enhance local targeting while minimizing systemic exposure (47). These approaches may offer a more balanced modulation of inflammation.

## 6 Future research directions

Several promising research directions emerge from current understanding of pyroptosis in COPD. Single-cell multi-omics approaches could comprehensively map pyroptosis activation patterns across different cell types and disease stages, identifying novel cell-specific therapeutic targets. Advanced organoid systems incorporating multiple cell types and mechanical stimulation may provide more physiologically relevant platforms for studying pyroptosis mechanisms and screening therapeutic candidates. Real-time visualization of pyroptosis in living systems represents another exciting frontier. The development of activatable fluorescent probes for gasdermin cleavage and pore formation could enable direct observation of pyroptosis dynamics in preclinical models and potentially in human studies using advanced imaging techniques. Such approaches would provide unprecedented insights into spatial and temporal patterns of pyroptosis activation.

Long-term safety assessment requires dedicated attention, particularly regarding cancer surveillance, infection susceptibility, and tissue repair capacity. Extended-duration preclinical studies and carefully designed clinical trials with comprehensive safety monitoring will be essential to fully characterize the risk profiles of pyroptosis-targeted therapies.

## 7 Conclusion

Pyroptosis has emerged as a critical mechanism driving the complex pathophysiology of COPD, representing a convergence point for multiple inflammatory pathways that contribute to disease progression. The sophisticated regulation of pyroptosis through canonical, non-canonical, and granzyme-mediated pathways demonstrates remarkable cell-type specificity and context-dependent activation, contributing to the heterogeneity that characterizes COPD clinical presentations.

The therapeutic landscape targeting pyroptosis continues to evolve, with multiple strategies showing promise at various stages of development. Inflammasome inhibitors offer upstream intervention points, while gasdermin-targeted approaches provide broader pathway coverage, and caspase inhibitors enable precise targeting of key executioners. Each strategy presents unique advantages and challenges that must be carefully considered in therapeutic development. Successful clinical translation will require thoughtful integration of multiple considerations, including optimal timing of intervention, sophisticated delivery systems, and appropriate combination with existing therapies. The development of reliable biomarkers for patient stratification and treatment monitoring will be essential for realizing the potential of precision medicine approaches in COPD management.

As research continues to unravel the complexities of pyroptosis regulation in COPD, this pathway offers exciting opportunities for developing novel treatments that address fundamental disease mechanisms beyond symptomatic management. Through continued investigation and thoughtful therapeutic development, pyroptosis-targeted strategies may eventually provide meaningful improvements in outcomes for patients suffering from this debilitating disease. The future of pyroptosis research in COPD lies in embracing the complexity of this cell death pathway while developing increasingly sophisticated approaches to modulate its activity in specific contexts and cell types. Such targeted interventions, guided by comprehensive biomarker profiles and advanced delivery systems, represent the next frontier in the ongoing effort to develop more effective treatments for COPD.
